# A Context-Aware, Computer-Vision-Based Approach for the Detection of Taxi Street-Hailing Scenes from Video Streams

**DOI:** 10.3390/s23104796

**Published:** 2023-05-16

**Authors:** Mahmoud Mastouri, Zied Bouyahia, Hedi Haddad, Leila Horchani, Nafaa Jabeur

**Affiliations:** 1 LARIA Research Unit, National School of Computer Science, Manouba University, Tunis 2010, Tunisia; mahmoud.mastouri@ensi-uma.tn (M.M.); zbouyahia@du.edu.om (Z.B.); 2 Computer Science Department, Dhofar University, Salalah 211, Oman; 3 CRISTAL-GRIFT Laboratory, National School of Computer Science, Manouba University, Tunis 2010, Tunisia; leila.horchani@ensi-uma.tn; 4 Computer Sciences Department, German University of Technology in Oman (GUtech), Muscat 1816, Oman; nafaa.jabeur@gutech.edu.om

**Keywords:** human–autonomous taxis interaction, explicit and implicit street-hailing recognition, deep learning, Robotaxis

## Abstract

With the increasing deployment of autonomous taxis in different cities around the world, recent studies have stressed the importance of developing new methods, models and tools for intuitive human–autonomous taxis interactions (HATIs). Street hailing is one example, where passengers would hail an autonomous taxi by simply waving a hand, exactly like they do for manned taxis. However, automated taxi street-hailing recognition has been explored to a very limited extent. In order to address this gap, in this paper, we propose a new method for the detection of taxi street hailing based on computer vision techniques. Our method is inspired by a quantitative study that we conducted with 50 experienced taxi drivers in the city of Tunis (Tunisia) in order to understand how they recognize street-hailing cases. Based on the interviews with taxi drivers, we distinguish between explicit and implicit street-hailing cases. Given a traffic scene, explicit street hailing is detected using three elements of visual information: the hailing gesture, the person’s relative position to the road and the person’s head orientation. Any person who is standing close to the road, looking towards the taxi and making a hailing gesture is automatically recognized as a taxi-hailing passenger. If some elements of the visual information are not detected, we use contextual information (such as space, time and weather) in order to evaluate the existence of implicit street-hailing cases. For example, a person who is standing on the roadside in the heat, looking towards the taxi but not waving his hand is still considered a potential passenger. Hence, the new method that we propose integrates both visual and contextual information in a computer-vision pipeline that we designed to detect taxi street-hailing cases from video streams collected by capturing devices mounted on moving taxis. We tested our pipeline using a dataset that we collected with a taxi on the roads of Tunis. Considering both explicit and implicit hailing scenarios, our method yields satisfactory results in relatively realistic settings, with an accuracy of 80%, a precision of 84% and a recall of 84%.

## 1. Introduction

Despite the uncertainty regarding their ability to deal with all real-world challenges, autonomous vehicle (AV) technologies have been receiving a lot of interest from industry, governmental authorities and academia [1]. Autonomous taxis (also called Robotaxis) are a recent example of such interest. Different companies are competing to lunch their autonomous taxis, such as Waymo (Google) [2], the Chinese Baidu [3], Hyundai with its Level 4 Ioniq 5s [4], NuTonomy (MIT) and the Japanese Tier IV, to mention a few. Robotaxis are not in the prototyping stage anymore, and different cities have regulated their usage on the roads, such as Seoul [5], Las Vegas [6], San Diego, and recently San Francisco, where California regulators gave Cruise’s robotic taxi service the permission to start driverless rides [7]. Uber and Lyft, two ride e-hailing giants, have partnered with AV technology companies to launch their driverless taxi services [8]. The Chinese DeepRoute.ai is planning to start the mass production of Level 4 autonomous taxis in 2024, to be available for consumer purchase afterwards [9]. In academia, recent research works have concluded that, besides the safety issue, the acceptance of Robotaxis by users is hugely affected by the user experience [10,11,12]. Researchers have identified different autonomous taxi service stages, mainly calling, pick-up, traveling and drop-off stages, and have conducted experiments to study the user experience during these different stages. Flexibility during the pick-up and drop-off stages is one of the issues raised by users. Currently, the users use mobile applications to “call” an autonomous taxi, and after boarding they interact either through a display installed in the vehicles or using a messenger app on their smartphones. One of the problems raised by participants is the difficulty of identifying the specific taxi they called, especially if there are many autonomous taxis around [10]. Mutual identification using QR code was inconvenient [10], and some participants suggested that other intuitive ways would be more interesting, such as hailing taxis through eye movement using Google glasses [12] and hailing gesture signals using smartwatches [12]. Participants also requested more flexibility with respect to the communication of the pick-up and drop-off locations to autonomous taxis. The current practice is to select pick-up and drop-off locations from a fixed list of stands, but users have the perception that taxis can be everywhere [10], and they expect autonomous taxis to be like traditional manned taxis in this respect [11].

These research findings show that scientists from different disciplines need to join their efforts to propose human–autonomous taxi interaction (HATI) models, frameworks and technologies that allow for developing efficient and intuitive solutions for the different service stages. In this paper, we address the case of one service stage that has been explored to a very limited extent, which is street hailing. The current state of the art reveals that only two works have studied taxi street hailing from an interaction perspective. The first work is the study of Anderson [13], a sociologist who explored traditional taxi street hailing as a social interaction between drivers and hailers. Based on a survey that he distributed to a sample of taxi drivers, he found that the hailing gestures used by passengers largely vary in relation to the visual proximity of the hailer to the taxi and to the speed at which the taxi is passing [13]. The second work, also from a social background, is the conceptual model proposed by [14] to design their vision of what would be humanized social interactions between future autonomous taxis and passengers during street-hailing tasks. Both works model taxi street hailing as a sequence of visually driven interactions using gestures that differ according to the distance between autonomous taxis and hailing passengers. Consequently, we believe that computer-vision techniques are fundamental in the development of automated HATI, particularly for the recognition of street-hailing situations. First, they are not intrusive, as they do not require passengers to use any device or application. Second, they mimic how passengers communicate their requests to taxi drivers in the real world, through visual communication. Third, like for manned taxis, they allow passengers to hail autonomous taxis everywhere without being limited to special stands [14]. Finally, they allow for better accessibility of the service, given that passengers who cannot/do not want to use mobile applications—for whatever reason—can still make their requests.

Apart from the work proposed in [15], our review of the state of the art revealed that there are no available automated solutions for taxi street-hailing recognition. In order to address this gap, in this paper, we propose a new method for the recognition of taxi street hailing using computer vision techniques. We report our preliminary results on the exploration of the role of the context in the detection of street-hailing scenes. In addition to the related state of the art, we conducted a survey in order to understand how 50 experienced taxi drivers in Tunis city (Tunisia) use contextual information to detect taxi street hailers. We distinguished between visual information and contextual information. The visual information captures the visual elements that taxi drivers rely on to identify explicit hailing behaviors, such as passenger gestures, relative position to the road and head orientation. Contextual information represents the non-visual information that taxi drivers rely on to recognize implicit hailing situations, such as time, space, etc. Elements of both visual and contextual information are included in a computer-vision pipeline that we designed to recognize taxi street hailers from video streams that were collected by capturing devices mounted on moving taxis. We tested our pipeline on a dataset collected by a taxi on the roads of Tunis, and our results are promising. Consequently, in this paper, we contribute with a new computer-vision-based method that uses visual and contextual information for the detection of both explicit and implicit hailing scenes from video streams.

The paper is structured as follows. In Section 2, we present a discussion of the related state of the art. In Section 3, we summarize the main findings from the taxi drivers’ survey regarding street-hailing detection and the role of visual and contextual information. In Section 4, we present our computer-vision-based approach for the detection of explicit and implicit street-hailing scenes. In Section 5, we present the training and test datasets that we used to experiment with our approach, along with the experimental settings. In Section 6, we present our quantitative and qualitative results and we discuss the strengths and weaknesses of our approach. We conclude in Section 7 by giving a brief overview of our future work.

## 2. Related Work

The state of the art on taxi street-hailing detection using computer vision techniques revealed the existence of only one work proposed by [15], which recognizes taxi street hailing from individual images based on the person’s pose and head orientation. We discuss this work in more detail in Section 6. Given the sparsity of research works about this specific topic, in the present section, we review the main works in the following related topics: (1) human–autonomous vehicle interaction (HAVI) and gesture recognition, (2) the detection of pedestrian intentions and (3) the identification of taxi street-hailing behavior.

### 2.1. Human–Autonomous Vehicle Interaction (HAVI) and Body Gesture Recognition

Since its introduction by W. Myron in 1991 [16], gesture detection and recognition have been widely used for the implementation of a variety of human–machine interaction applications, especially in robotics. With the recent technological progress in autonomous vehicles, gestures have become an intuitive choice for the interaction between autonomous vehicles and humans [17,18,19]. From an application perspective, gesture recognition techniques have been used to support both indoor and outdoor human–autonomous vehicle interactions (HAVIs). Indoor interactions are those between the vehicles and the persons inside them (drivers or passengers). Most of the indoor HAVI applications focus on the detection of unsafe driver behavior, such as fatigue [20] and on vehicle control [21]. Outdoor interactions are those between autonomous vehicles and persons outside them, such as pedestrians. Most of the outdoor HAVI applications focus on the car-to-pedestrian interaction [22,23] and car-to-cyclist communication [24] for road safety purposes, but other applications have been explored, such as traffic control gestures recognition [25], where traffic control officers can request an autonomous vehicle to stop or turn with specific hand gestures.

From a technological perspective, a lot of research work has been conducted on the recognition of body gestures from video data using computer-vision techniques. Skeleton-based recognition is one of the most widely used techniques [26], both for static and dynamic gesture recognition. A variety of algorithms have been used. Traditional techniques include Gaussian mixture models [27], recurrent neural network (RNN)with bidirectional long short-term memory (LSTM) cells [28], deep learning [29] and CNNs [30]. The current state of the art for indoor and outdoor gesture recognition builds on deep neural networks. A recent review of hand gesture recognition techniques in general can be found in [31,32].

### 2.2. Predicting Intentions of Pedestrians from 2D Scenes

The ability of autonomous vehicles to detect pedestrians’ road-crossing intentions is crucial for their safety. Approaches of pedestrian intention detection can be categorized into two major categories. The first category formalizes intention detection as a trajectory prediction problem. The second category considers pedestrian intention as a binary decision problem.

Several models and architectures have been developed and deployed, aiming at achieving a high-accuracy prediction of pedestrian intention using a binary classification approach. Unlike other methods, binary classification utilizes different tools and techniques depending on the data source and the data characteristics and features. For instance, the models based on RGB input use either 2D or 3D convolutions. In 2D convolution, a sliding filter is used along the height and width, and in 3D settings, the filter slides along the height, width and temporal depth. Using 2D convolutional networks, the information is propagated across time either via LSTMs or feature aggregation over time [33]. For instance, the authors in [34] proposed a two-stream architecture that takes as input a single excerpt from typical traffic scenes bounding an entity of interest (EoI) corresponding to the pedestrian. The EoI is processed by two independent CNNs producing two feature vectors that are then concatenated for classification. Authors in [35] presented an extension of these models by integrating LSTMs and 3D CNNs, and those in [36] did so by feeding many frames into the future and carrying out the classification using these frames.

Other methods that use the skeletal data extracted from the frames have been proposed. These methods directly operate on the skeleton of the pedestrians. The main advantage of these methods is that the data dimensions are significantly reduced. The yielded models are therefore less prone to overfitting [37]. Recently, a new method was proposed based on the individual keypoints in order to achieve the prediction of pedestrian intentions but from a single frame. Another method proposed by [38] exploits contextual features, such as the distance separating the pedestrian from the vehicle, his lateral motion, and his surroundings as well as the vehicle’s velocity as input to a CRF (conditional random field). The purpose of this model is to predict in an early and accurate fashion pedestrian’s crossing/not-crossing behavior in front of a vehicle.

### 2.3. Identification of Taxi Street-Hhailing Behavior

The topic of recognizing taxi street hailing has been studied by sociologists in order to explore how taxi drivers perceive and culturally interact with their environment, including passengers [39]. An interesting work is the work of Anderson, who studied gestures, in particular, as a communication channel between taxi drivers and passengers during hailing interactions [13]. Based on a survey that he distributed to a group of taxi drivers in San Francisco, CA, USA, the researcher wanted to explore how taxi drivers evaluate street hails in terms of clarity and propriety. Clarity refers to “the ability of clearly recognizing a hailing behavior and distinguishing it from other one”, such as waving to a friend. Propriety refers to“the ability to identify if the passenger can be trusted morally and socially” so the taxi driver can decide either to accept the hailing request or not [13]. In the context of our work, it is the clarity aspect that is more relevant, and with this respect, Anderson’s results are interesting. He found that the method of hailing adopted varies largely in relation to the visual proximity of the hailer to the taxi, and to the speed at which the vehicle is passing [13]. When the driver and the hailer are within range of eye contact, the hailer “can use any waving or beckoning gestures to communicate his intention, such as raising one’s hand, standing on the curb while sticking arms out sideways into the view of the oncoming driver” [13], etc. However, if the hailer and the driver are too far from each other, “hailers need to make clear that they are hailing a cab as opposed to waving to a friend, checking their watch, or making any number of other gestures which similarly involve one’s arm” [13]. Taxi drivers who participated in the survey specified that the best and most clear gesture, in this case, is the “Statue of Liberty”, where the“hailer stands on the curb, facing oncoming traffic, and sticks the street-side arm stiffly out at an angle of about 100–135 degree” [13]. However, taxi drivers pointed out that there are many other hailing gestures, and they may even depend on the hailers’ cultural backgrounds. Similarly, the conceptual model of human–taxi street-hailing interaction proposed in [14] assumed that, depending on the distance between taxis and passengers, different hailing gestures can be used, such as waving or nodding.

Both works stressed that passengers need to use explicit gestures in order to communicate their desire to ride a taxi to the drivers. The explicit gestures may vary according to the proximity between the passengers and the taxis, as well as according to the cultural background, but hand waving remains the most common hailing gesture. Nevertheless, in many real cases, and for different reasons, passengers may not be able to make explicit distinctive gestures. For example, a person who is standing on the curb of the street, holding bags in both hands and looking towards vehicles could be a potential taxi hailer. This could be confirmed by real-life observations of several cases, where vacant taxi drivers slow down after perceiving pedestrians standing/walking on the roadside, even though these pedestrians did not make any explicit hailing gestures. This is simply because taxi drivers consider these pedestrians to be potential street-hailing passengers, and they slow down to confirm or dispel their intuition. Consequently, there is a need to propose a method that can detect both explicit and implicit taxi street-hailing behavior from traffic scenes. For this purpose, we propose to combine both visual and contextual information in one computer-vision-based approach. To the best of our knowledge, the integration of visual and contextual information for the detection of both explicit and implicit street-hailing behavior has not been the main subject of any previous research work.

## 3. Visual and Contextual Information

One of the main concerns that motivated our work is how taxi drivers recognize a street hailing. To address this, we designed a survey that we used to interview a group of 50 experienced taxi drivers in the city of Tunis, the capital of Tunisia. Our initial results showed that, in general, taxi drivers use two types of information, visual and contextual information, respectively.

**Figure 1:**
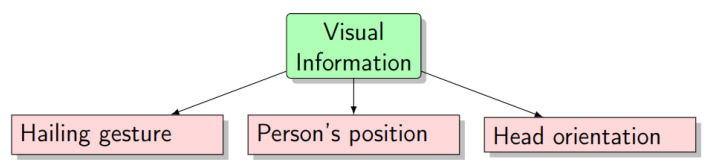
Visual information.

The visual information refers to the aspects that a taxi driver can visually verify to recognize any explicit taxi street-hailing scene while he is cruising the road network. All taxi drivers agreed that an obvious street-hailing scene is composed of a person who is standing on the roadside, looking towards the taxi and waving with his/her hand. Consequently, we decomposed the visual information of a street-hailing scene into three elements: (1) the presence of the hailing gesture, (2) the position of the person relative to the road, and (3) the orientation of the person’s head (Figure 1). The combination of these three elements allows for the estimation of the confidentiality of street-hailing detection. For example, a person who is standing far from the roadside, waving his/her hand and looking in the opposite direction of the taxi would have a low probability of corresponding to a street-hailing case. However, if the person is looking towards the taxi, the probability of having a street-hailing case will increase.

In case all three visual information elements are present, the scene is automatically interpreted as an explicit street-hailing case, and there is no need to check other types of information. However, if one of the three visual information elements is not detected, there is a need to evaluate other pieces of information (that are not visual) in order to evaluate the presence of street hailing. We collectively refer to these non-visual pieces of information as contextual information.

**Figure 2:**
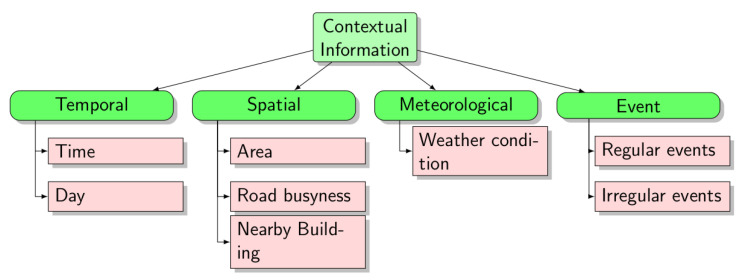
Contextual information.

Contextual information corresponds to a kind of external knowledge (external to the visual hailing scenes) that taxi drivers often use to recognize implicit street-hailing scenes, i.e., cases where passengers do not use explicit hailing gestures. In order to identify the list of potential contextual information elements, we started by reviewing related works that studied the strategies used by taxi drivers to search for potential passengers [40,41,42,43,44]. We also reviewed some works about the factors that affect the variability and prediction of taxi demand [45,46,47,48]. We assumed that these factors could be good candidates for contextual information. For example, authors in [46] concluded that there exists a correlation between taxi demand prediction and temporal, spatial, weather and special events factors. The list of contextual information elements that we combined from the state of that art is illustrated in Figure 2. The taxi drivers that we interviewed collectively confirmed the same aspects.

Based on our survey, we concluded that taxi drivers recognize street hailing using both visual and contextual information according to a two-step reasoning process. In the first step, they evaluate the existence of the three elements of the visual information, i.e., the hailing gesture, the person’s relative position to the road and the head orientation. If the three elements are present, an explicit street-hailing case is automatically detected, and there is no need to use further information. However, if one or more elements of the visual information is/are not confirmed, a second step is required, which consists of evaluating elements of contextual information to have better judgment. In case enough elements of contextual information are detected, an implicit street-hailing case is detected; otherwise, no street hailing is recognized.

In this paper, we propose to implement a computer-vision-based pipeline for taxi street-hailing detection that mimics the two-step reasoning process used by taxi drivers. We believe that it is important to consider both explicit and implicit street-hailing cases. Explicit cases can be relatively well described using the three elements of information that we identified. Implicit cases are more challenging to detect, and we believe that the use of contextual information could be helpful. This could accommodate real situations, where passengers are not able to make explicit hailing gestures (for any reason) but still desire to hail a taxi. In the next section, we present our proposed computer-vision-based method for the detection of both explicit and implicit taxi street-hailing cases.

## 4. Proposed Method

In this section, we present the new method that we propose to recognize street-hailing cases from video sequences. The method comprises three stages. The first stage consists of detecting the objects of interest (persons and road segments) from the sequences. The second stage consists of detecting the visual information components presented in the previous section, i.e., the hailing gesture, the position of the detected person(s) relative to the road, and the orientation of the potential passengers’ heads. The third stage consists of the integration of the contextual information, if needed, in order to have a final decision about whether street hailing is detected or not.

### 4.1. First Stage: Detection of Objects of Interest

**Figure 3:**
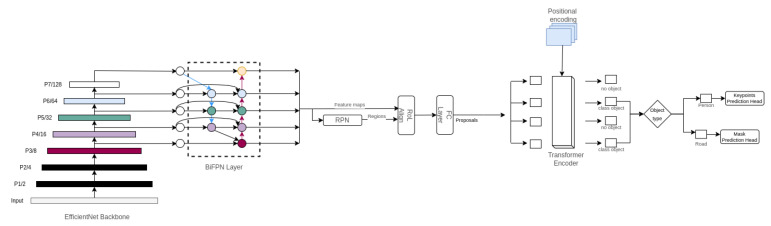
Network architecture of the first stage.

The first stage consists of detecting the persons and the road segments from the video data, and is implemented as a neural network architecture (Figure 3) that takes an image (frame) in the input and returns the road segmentation mask and the persons’ keypoints in the output. It is an end-to-end model to extract the keypoints masks for persons and the mask map for the road. The architecture that we used is based on the transformer-based set prediction with RCNN (TSP-RCNN) proposed by [49], which solves the slow convergence problem of the detection transformers (DETR) [50].

In the following, we present each architectural choice of the proposed method TSP-RCNN and we explain how we adapt it to our requirements.

Elharrous and his team [51] presented a comprehensive study that compares the more efficient and accurate feature extractors in all deep learning tasks, including the computer vision ones. EfficientNet-B3 + BiFPN [52] and ResNet-50 + FPN are two of the most widely used backbones. EfficientNet-B3 + BiFPN has superior precision, computational cost, and inference latency. Since our project requires detecting large-scale objects (roads) and small objects (crowded persons), we opted for utilizing the EfficientNet-B3 backbone with bi-directional FPN (BiFPN), which provides a feature extraction procedure that extends to both small and large objects.

The proposed method is based on TSP-RCNN [49], which uses an RCNN mechanism plus the intrusion of a transformer decoder to generate the class object boxes while changing the backbone to Efficient-B3 + BiFPN based on the EfficentDet architecture and adding multi-head for each of the detection and segmentation tasks.

### 4.2. Second Stage: Extraction of the Visual Information

After extracting the persons and road segments in the first stage, the second stage consists of extracting the person’s position estimation, head direction, and hailing gesture detection from consecutive frames.

#### 4.2.1. Person’s Position Estimation

In general, a person’s relative position estimation is not trivial. However, after the extraction of the persons and road segments in the first stage, we can estimate if a detected person is located on the side of the road, on the road, or away from both.

Let *K* be a set of *n* keypoints of a given person, where $k_{i\in [0\ldots n-1]}=\left(x_{i},y_{i}\right)$, $x_{i}$ and $y_{i}$ each represents the point’s position in an image of width *w* and height *h* (w×h).

The standing position of the person is a circle φ with a radius *r* and origin *I*, where

(1) $$\begin{array}{c} \left\{\begin{array}{c} I=k_{{\mathrm{argmax}}_{i}y_{i}}\\ r=\max\left(d\left(k_{i},k_{j}\right)\right),i,j\in \left[0..n-1\right]\\ d=\frac{\sqrt{{\left(x_{1}-x_{2}\right)}^{2}+{\left(y_{1}-y_{2}\right)}^{2}}}{2} \end{array}\right. \end{array}$$

The circle’s origin *I* is the closest point of the detected person to the ground. The farthest two points represent the circle’s diameter and the person’s distance from the camera.

Let us consider *R* a set of *m* points (pixels) of the detected road, where $R_{i\in [0..m-1]}=\left(x_{i},y_{i}\right)$, $x_{i}$ and $y_{i}$ each represents the pixel position in an w×h image.

The detected person is

(2) $$\begin{array}{c} \left\{\begin{array}{c} \mathrm{on}\;\mathrm{the}\;\mathrm{road}\;\mathrm{if}\;R\cap \varphi =\varphi \\ \mathrm{on}\;\mathrm{the}\;\mathrm{roadside}\;\mathrm{if}\;R\cap \varphi \neq \varphi \;\mathrm{and}\;R\cap 2\varphi \neq \emptyset \\ \mathrm{away},\;\mathrm{otherwise} \end{array}\right. \end{array}$$

We assume that a person who is looking for a taxi should be very close to the road. Consequently, we interpret a detected person as a potential street hailer if they are not very far from the road.

#### 4.2.2. Person’s Head Direction

Let us consider the set {A,B,C}∈K, with *K* being the set of *n* keypoints of a given person defined in Section 4.2.1, where *A*, *B* and *C* respectively correspond to the left eye, right eye, and nose if detected, and otherwise to the right shoulder, the left shoulder, and head if detected.

The head direction is a vector $\vec{D}$ defined as follows:

(3) $$\begin{array}{c} \left\{\begin{array}{c} \vec{U}=\left(B_{x}-A_{x},B_{y}-A_{y}\right),\vec{U}=\left(C_{x}-C_{x},B_{y}-A_{y}\right)\\ \vec{D}=\vec{U}\times \vec{V} \end{array}\right. \end{array}$$

#### 4.2.3. Person Tracking and Hailing Gesture Detection

Let us recall that the input of our method is a video sequence composed of a set of frames. Hailing is a dynamic scene in which the same person is performing waving gestures over a consecutive set of frames. Consequently, before we detect the waving gesture, we need to track the identical person(s) over the sequence of frames using object-tracking techniques. In our method, we implemented object tracking by simply checking if a person $p^{t}$ in the frame *t* has any corresponding elements in the frame t+1 by using a Hungarian algorithm. The score assignment for the Hungarian algorithm can be established by IoU (intersection over union), meaning that if the bounding box overlaps with the previous one, we conclude that it is probably the same person.

To detect the hailing gesture, we analyzed multiple videos of a person performing hand-hailing movements. After running several hailing examples, we noticed that the following four key points can disguise the specific hand movement: shoulder, elbow, wrist, and hip.

**Figure 4:**
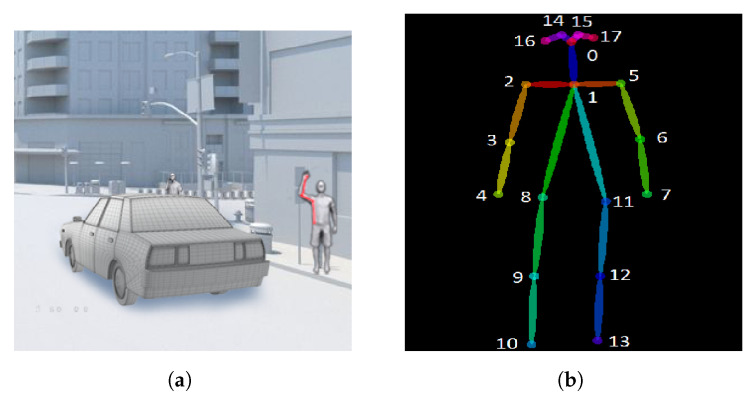
Hailing Example showing (**a**) Focus key points and (**b**) Human body key points.

Figure 4a illustrates the area we focus on during the hailing detection, and Figure 4b shows the key points map of a human body and their respective identifiers.

Finally, we proposed a key points angle approach defined as follows:

- Let A, B, C, and D be the key points for the shoulder, elbow, wrist, and hip, which respectively correspond to key points numbers 2, 3, 4, and 8 in Figure 4b, for the right side and numbers 5, 6, 7 and 11 for the left side, respectively.
- We define $\theta_{1}$ as the angle between hip, shoulder and elbow, so $\theta_{1}=D\hat{A}C$.
- We define $\theta_{2}$ as the angle between shoulder, elbow, and wrist, so $\theta_{2}=A\hat{B}C$ where the angle between 3 points in 2-dimensional space is defined as follows:
  – $\theta =A\hat{B}C$
  – $rad_{\theta }=\arctan2\left(C_{y}-B_{y},C_{x}-B_{x}\right)$, arctan2 is the element-wise arc tangent of $\frac{x_{1}}{x_{2}}$ choosing the quadrant correctly.
  – $A\hat{B}C=\left|\frac{rad_{\theta }\times 180}{\pi }\right|$

Following this strategy, we then measure the two angles in each frame of a clip of a hailing person twice, one for his right hand and the other for his left hand.

**Figure 5:**
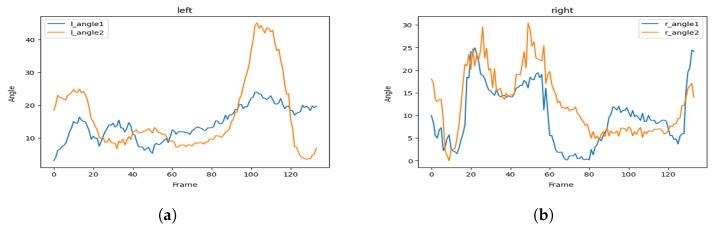
Hailing example: angles variation of (**a**) the left side and (**b**) the right side.

**Figure 6:**
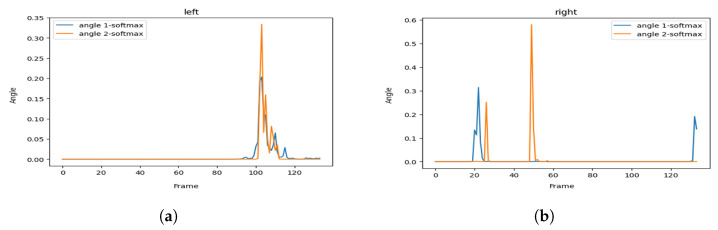
Hailing example: smoothed (soft-max) angles variation for (**a**) the left side and (**b**) the right side.

Figure 5 represents the visualization of the variation of the angles, and Figure 6 represents the visualization of the variation of the angles after applying soft-max in order to smooth it.

As we can see, the hailing movement is very clear after applying smoothness to the variation of the angles. In the left side of Figure 6, we can identify a hailing movement between the 90th frame and the 110th frame. By applying the same logic, we can identify a hailing on the right side between the 15th frame and the 30th frame.

### 4.3. Final Stage: Scoring and Hailing Detection

In this section, we explain how street-hailing cases are detected based on a scoring scheme that integrates the extracted visual information with the contextual information.

As we explained in Section 3, the interviews of taxi drivers show that street hailing can be explicit or implicit. Explicit hailing is mainly detected based on the visual information (a person waving on the road to stop a taxi); otherwise, implicit hailing is evaluated relying on contextual information. Consequently, we designed the final stage of street-hailing detection using the following decision flow:

(1) Elements of the explicit street hailing are evaluated first, and if they are all detected, a street-hailing case is recognized.
(2) If one or more elements of the visual information is/are not detected, contextual information is used in order to evaluate the existence of implicit street hailing.

The decision flow is implemented using the following scoring scheme relying on two main components: visual and contextual information.

Visual information:

The three components of the visual information are scored as follows:

– Hailing gesture is scored as $\alpha_{1}$ if detected and zero otherwise.
– The score of the standing position is $\beta_{1}$ if the person is on the side road, $0.5\beta_{1}$ if the person is close to the side road (e.g., on the road), and zero otherwise.
– The score of the head direction is $\gamma_{1}$ if the person is looking toward the road or the taxi and zero otherwise.

The variables $\alpha_{1}$, $\beta_{1}$, and $\gamma_{1}$ represent a scoring system of 100 points, where if one of these variables is absent, then the score never reaches 90, and hence the contextual information is used for further evaluation.

Contextual information:

– Spatiotemporal information: $\alpha_{2}$ can go as high as 60 points in the case of a high-demand area and time and as low as zero contrariwise.
– Meteorological information: $\beta_{2}$ can go as high as 30 points in the case of very bad weather and as low as zero contrariwise.
– Event information: $\gamma_{1}$ if there an event in the corresponding place–date and zero otherwise.

The scoring system works as follows:

– The maximum score is 100 points.
– If the visual information reaches θ = 90 points, the person is classified as a street hailer, and no contextual information is used.
– Otherwise, the contextual information is used as a percentage of the difference between 100 and the visual information points, e.g., if the visual information is 60 points and the contextual information is 50 points, the score is 60+(100−60)×50%=80 points.

## 5. Dataset and Experimental Settings

We implemented the proposed method using the PyTorch deep learning library, OpenCV computer vision library, and Numpy. Python was selected because it is suitable for deep learning and computer vision tasks, is supported in multiple platforms and is capable of handling real-time computer vision tasks.

### 5.1. Datasets

**Figure 7:**
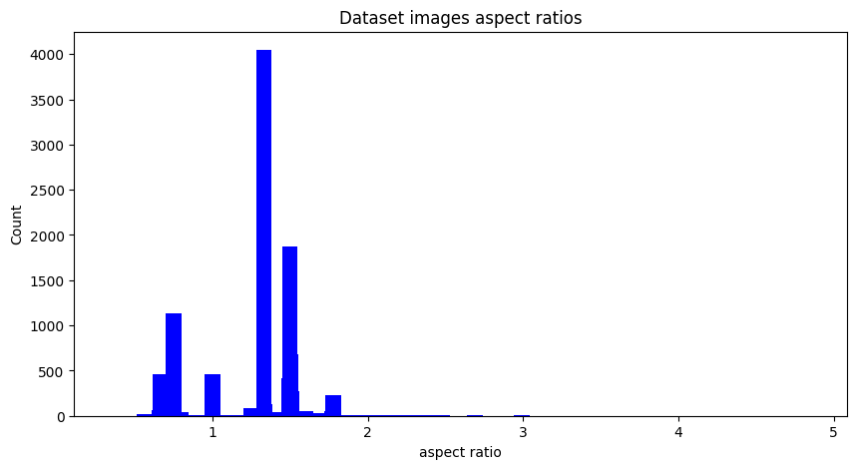
Data set aspect ratio.

For the training part, we used the Common Object in Context (COCO) dataset [53], a large-scale object detection, segmentation, and captioning dataset that is the benchmark for most state-of-the-art methods in various computer vision tasks. Since our task is not a general-purpose detection/segmentation task, we sub-sampled the COCO-stuff dataset into 15,402 images containing the road and person categories. We found that the average height and width of images are 477.23 and 587.85, respectively (Figure 7). The 587.85 aspect ratio is 1.23, which is fine to use with a non-destructive resize and with a padding approach to resize our images to the desired size suitable for our backbone input.

For the test dataset, we opted to mix (1) the previously presented COCO dataset, excluding the part we used for the training, and (2) a set of images that we filmed by ourselves on the roads of Tunis city, Tunisia.

**Figure 8:**
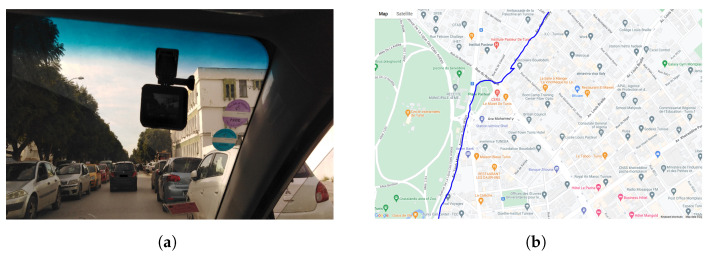
Recording settings showing (**a**) the camera mounting and (**b**) an example of position recording.

We mounted a Go-Pro Hero 10 camera on the front glass of a probe taxi, on the passenger side facing the road and the sidewalk at the same time to capture pedestrians and roads, as shown in Figure 8a. The recording process took place at different times and locations in the Tunisian capital with a constant resolution of 1080 × 1920 using 120 frames per s. The purpose was to capture different scenes at different spatial–temporal moments corresponding to both high and low taxi street demand (crowded vs. uncrowded areas).

The data collection trips were tracked using a GPS-tracking mobile application, so all the collected video sequences are time- and GPS stamped (Figure 8b). The GPS trajectories were then augmented with other contextual information using Google Maps, such as temperature, the busyness of the roads and special events, if any.

Finally, we filtered the collected data, and we selected only the best clips from the recorded videos. We obtained about 3 h of good-quality clips that we used as a test dataset. More data are being collected to build a complete dataset that can be used for training, validation, and testing.

**Figure 9:**
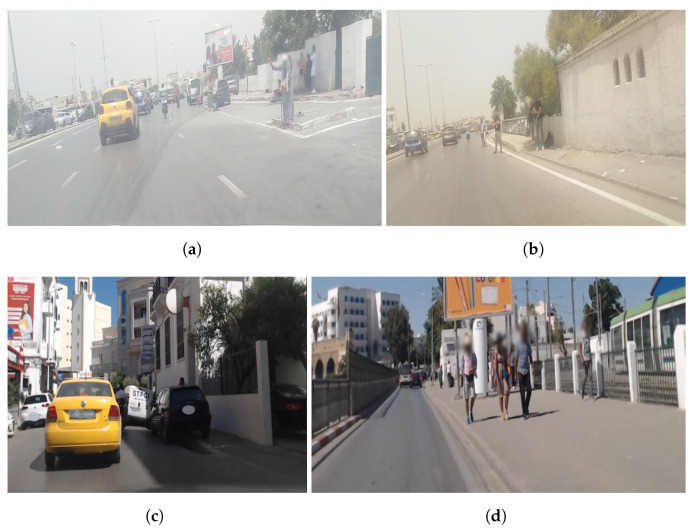
Examples from the test dataset showing different situations of taxi street-hailing contexts including (**a**) a crowded area, (**b**) an area with few cars and people, (**c**) a secondary road with no people and (**d**) and an area with many people and far away cars.

Figure 9 shows a collection of images that we captured: Figure 9a shows an area with high taxi demand (Beb Alioua, Tunis) with many people and cars; Figure 9b shows the same area but at a different location with fewer cars and people; Figure 9c shows an area with high demand (Monplaisir, Tunis) but in a secondary road such that we have many cars and no people; and Figure 9d shows very visible people and far away cars. The objective is to capture different situations that will help us evaluate our model in extracting many anchor sizes for persons and roads and to make sure it will work in various scenarios. 

### 5.2. Experiment Settings

For the training parameters, we used the same training process for the proposed TSP-RCNN method since it is our base model.

As mentioned in Section 4.1, we used the EfficientNet-B3 backbone, a weighted bi-directional feature pyramid network as described in [52], where the output of BiFPN is passed to the RPN selecting 700 scored features. The RoI Align operation and a fully connected layer were applied to extract the proposed features from the ROIs. We used a 6-layer transformer encoder of width 512 with 8 attention heads. The hidden size of the feed-forward network (FFN) in the transformer was set to 2048. Finally, for the detection heads, we used the default training parameters in detectron2 [54], as we used the default mask head for road segmentation and the pre-trained keypoints head for keypoints detection.

Unlike the multi-task end-to-end networks, e.g., HybridNets [55] and YOLOP [56] which use Dice and Focal losses, we used only Focal loss, as it is an object detection problem for the first stage of the network.

Following TSP-RCNN, we used AdamW to optimize the transformer, and SGD with a momentum of 0.9 to optimize the other parts with a 3× scheduler.

As evaluation metrics, we used the average precision (AP) and recall IoU (%) as primary metrics and FLOPs for the number of computations. All are provided by Detectron2.

## 6. Experimental Results and Discussion

In order to evaluate the performance of the detection of objects of interest, we followed the same training protocol used in the TSP-RCNN model, which is described in [49], and we utilized a combination of L1 and Generalized IoU losses for regression. Focal loss is used for weighting positive and negative examples in classification for both TSP-FCOs and TSP-RCNN.

To evaluate the performance of our proposed method for street taxi-hailing detection, we calculated the accuracy, precision, recall and specificity metrics based on the calculated values of true positive (TP), true negative (TN), false positive (FP), and false negative (FN) rates, as follows:

- Accuracy: It measures the overall correctness of the model’s predictions and is defined by the following:
  $$\frac{TP+TN}{TP+TN+FP+FN}$$
- Precision: It measures the proportion of positive predictions that are correct, and is defined by
  $$\frac{TP}{TP+FP}$$
- Recall (also known as sensitivity): It measures the proportion of actual positive cases that were correctly identified by the model. It is defined by
  $$\frac{TP}{TP+FN}$$
- Specificity: It measures the proportion of actual negative cases that were correctly identified by the model. It is defined by the ratio
  $$\frac{TN}{TN+FP}$$

### 6.1. Detection of Objects of Interest

**Figure 10:**
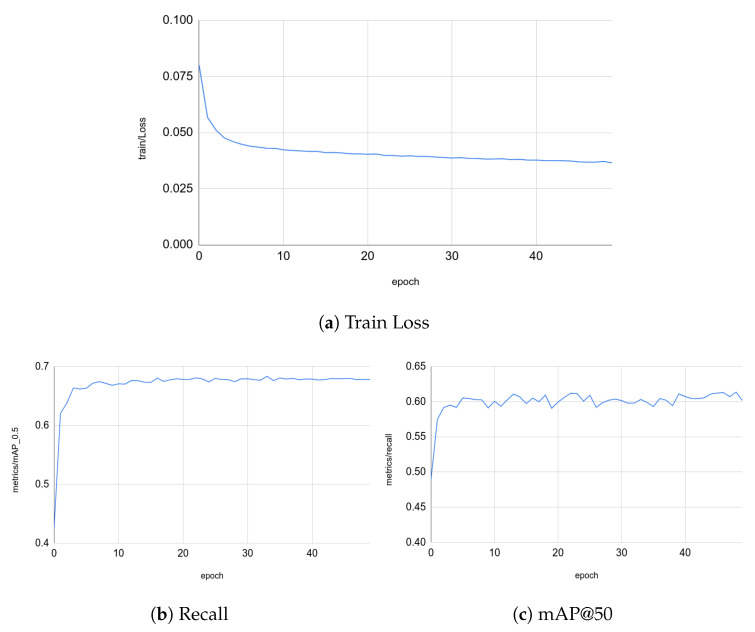
Training results.

With respect to the results of this training process, we tackled only the accuracy of the bounding boxes at the end of the transformer encoder since we are using pre-trained head keypoints. As illustrated in Figure 10, the precision is 68%, and the recall value is 60%. These values are acceptable with respect to the COCO dataset [57].

**Figure 11:**
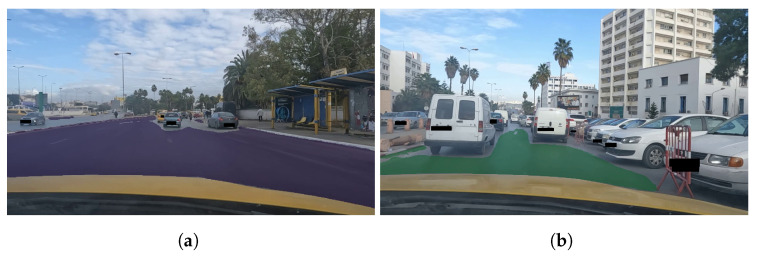
Road segmentation results of (**a**) example 1 and (**b**) example 2.

Figure 11 illustrates the results of two different examples of the road-segmentation tasks. The image highlights the pixels of the road and manages to distinguish the road from the sidewalk. However, the segmentation does not extend to the far-away road, which will not affect our detection since the proposed method works on video streams and the far-away road will eventually become the up-front road for the recording camera.

**Figure 12:**
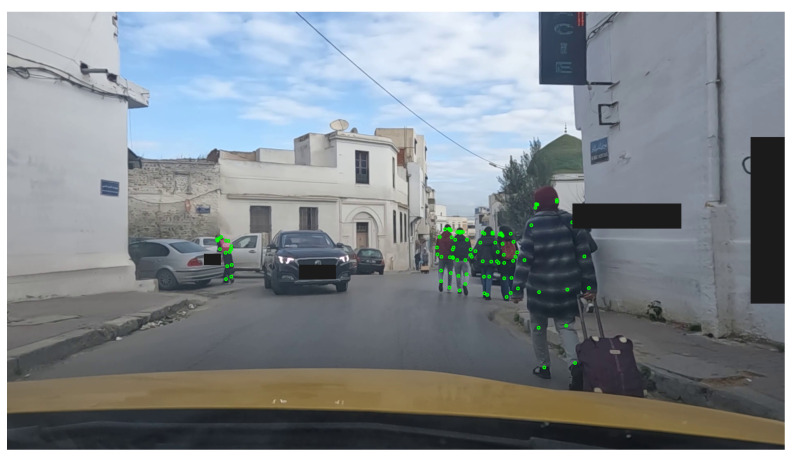
People keypoint detection.

Lastly, Figure 12 illustrates an example of human keypoints detection results. Our model succeeds in detecting the keypoints of a crowded area (people very close to each other) and since the keypoints detection plays a major role in the second stage, we used a higher confidence level of 70%.

### 6.2. Detection of Street Hailing

In our proposed architecture, we used an unsupervised method for the detection of street hailing, which does not require a training process. However, given that we used a dataset that we collected as a test set, we evaluated the performance of the overall street-hailing detection process (including both explicit and implicit) using true positive (TP), true negative (TN), false negative (FN) and false positive (FN) metrics. Once a street-hailing detection was carried out, we evaluated the ability of the proposed method to discern the type of hailing (explicit or implicit). In this case, we used only the false negative and true positive metrics since the existence of a street-hailing action was already confirmed. Table 1 reports the accuracy, precision, recall and specificity scores of the obtained results. Our method achieved an accuracy of 80% and, most importantly, a precision and recall of 84%, which implies that the method can correctly, in most cases, identify both explicit and implicit street hailing.

In the following examples, we depict four scenarios corresponding to explicit street hailing, implicit street hailing, no street hailing, and undetected street hailing, respectively. Each of these examples illustrates qualitatively the performance of the proposed method in various contexts.

#### 6.2.1. Example 1: Explicit StreetHailing

**Figure 13:**
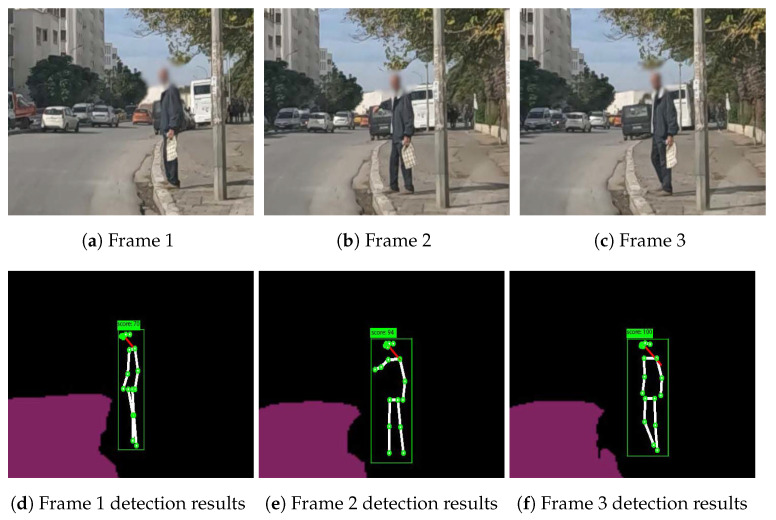
Example 1: Explicit hailing.

**Figure 14:**
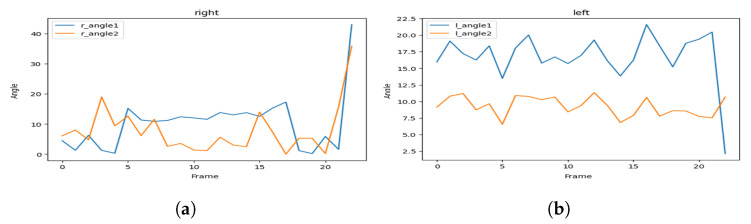
Non-smoothed angles variation of (**a**) the right side and (**b**) the left side.

**Figure 15:**
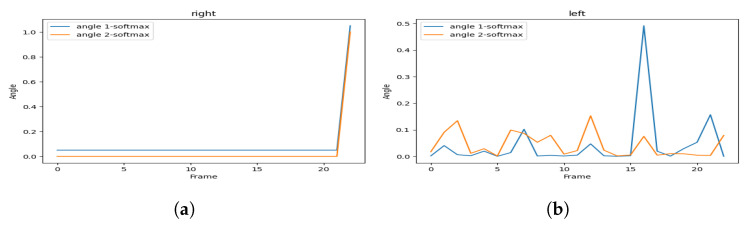
Smoothed (soft-max) angles variation of (**a**) the right side and (**b**) the left side.

In Figure 13, we illustrate an example of an explicit hailing scenario captured from a 20-frame scene (we display only 3 due to the page width limit). Figure 14 displays the angle variation of the person performing the hailing without smoothing out the values, and Figure 15 illustrates the same variation with soft-max as the smoothing function.

These visualizations show a simultaneous peak (Figure 15a) in the tracked angles between the shoulder, elbow, wrist, and hip, similar to the one in Figure 6a that represents, in most cases, a hand-waving action (which can be interpreted as a hailing action), different from the one in the next example.

Table 2 illustrates the score of the visual information corresponding to the explicit hailing case, which is 100 points, given that all the elements of visual information are detected. Consequently, the person is flagged as a street-hailing case and no contextual information is used (Figure 13d–f).

#### 6.2.2. Example 2: Implicit Street Hailing

**Figure 16:**
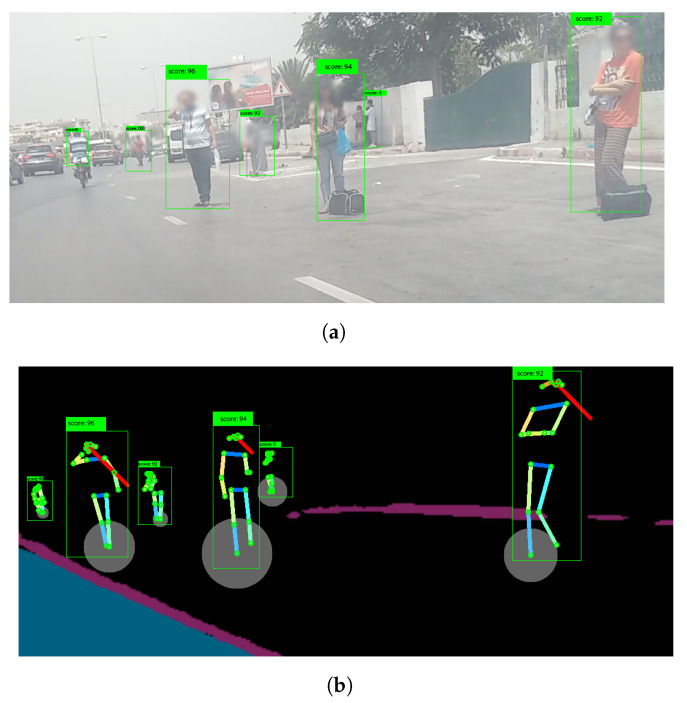
Example 2: Implicit hailing illustrating (**a**) a scene and (**b**) its visual detection.

**Figure 17:**
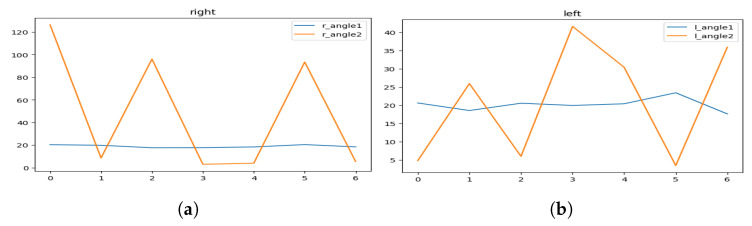
Example 2: Non smoothed angles variation of (**a**) the right side and (**b**) the left side.

**Figure 18:**
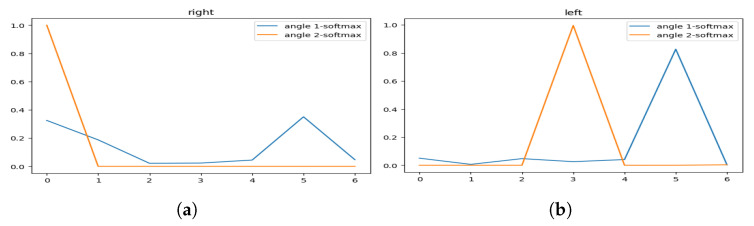
Example 2: Smoothed (soft-max) angles variation of (**a**) the right side and (**b**) the left side.

Figure 16a illustrates an example of a potential implicit street-hailing scene. We can recognize three people close to the taxi’s camera, all of them in an idle position, standing by the roadside. The front two people present no hand movement, but the third shows a hand movement (compared to previous frames) which is not recognized as a hailing action because his hand is on his head. As shown in Figure 17 and Figure 18, for the person resting his hand on his head (left), there is no simultaneous peak in the angle variation, even though the action is similar to hand waving or hand hailing.

Table 3 illustrates the obtained visual and contextual information scores corresponding to the case illustrated in Figure 16 (the person at the left). The visual information score does not reach the 90 points threshold, and therefore, contextual information is used. Given that the scene was captured in Beb Alioua (an area with high taxi demand) at 11:00 a.m. (peak time) and with a temperature of 35 °C in the summer, the score of the contextual information is 90 points as shown in the same table. The final score is 60+(100−60)×90%=96 points, and the person is considered a street-hailing passenger.

#### 6.2.3. Example 3: No Street Hailing

**Figure 19:**
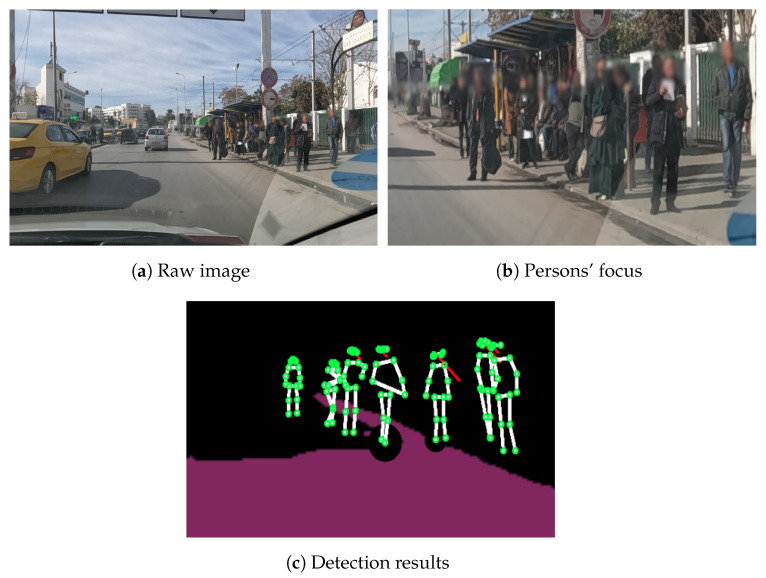
Example 3: No street hailing case illustrating (**a**) a raw image, (**b**) the persons’ focus and (**c**) the corresponding detection results.

In the example illustrated in Figure 19, we present a situation of no street hailing. It is a crowded area, but there is no overlap with people. Individuals are accurately detected, and after tracking each one, we can see that few of them make good candidates for taxi street hailing from the visual information perspective (the people are sitting, not looking at the road/taxi, etc.). The contextual information also does not reveal a sufficient score since this is a bus station where passengers are waiting for the bus. Consequently, no street hailing is detected.

Table 4 illustrates the obtained visual and contextual information scores corresponding to the case illustrated in Figure 19. The visual score for the persons in this scene is 30 points for the standing position, 10 points for the head direction, and 0 for the hailing gesture, with a total of 40 points. The contextual information score has 30 points for the spatial–temporal parameter given that the time corresponds to a peak interval but the location corresponds to a low-demand area. The weather information achieves 10 points because of the heat, while no event is detected in the area. The contextual information score is equal to 40 points, and the final score is =40+(100−40)×40%=65 points, and consequently no street hailing is detected.

#### 6.2.4. Example 4: Undetected Street Hailing

**Figure 20:**
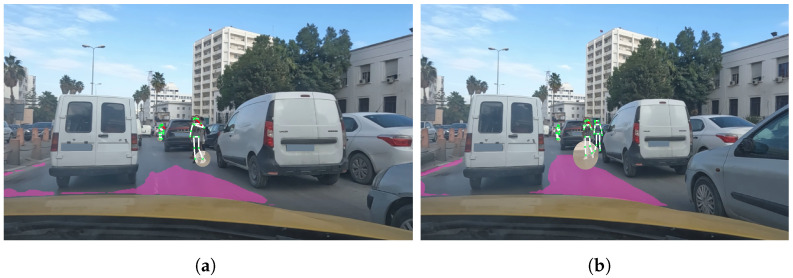
Example 4: Undetected street hailing illustration with 2 frames (**a**,**b**).

In the example illustrated in Figure 20, we present another scenario, where our method fails to detect a hailing case. As illustrated in Figure 20a,b, the frames in the observed scene show a crowded environment with overlapping between the pedestrians and vehicles, which hides crucial body parts required for the keypoints detection. Therefore, the accurate tracking of keypoints is impeded. This scenario illustrates the limitations of using pure computer vision methods for the detection of street hailing.

### 6.3. Discussion

As we previously mentioned, taxi street hailing has been explored to a very limited extent in the state of the art. To the best of our knowledge, the only automated solution for taxi street-hailing recognition is the computer-vision-based approach proposed in [15] to detect car street-hailing intentions from individual images. Even though we share the common motivation that computer-vision-based car-hailing recognition is more convenient for autonomous taxis than traditional online booking methods, our approach is fundamentally different with respect to two main perspectives. The solution proposed in [15] detects the street-hailing intention from individual images by fusing two main elements: (1) the hailing pose of the person and (2) the attention of the person to the taxi (expressed by the eyes’ orientation towards the taxi). In simple words, a person who is making the hailing pose and looking towards the taxi potentially has the intention of hailing. By considering only individual images, the temporal dynamic hailing scenes (such as waving hands) are not considered, and this is the reason why the authors talk about the hailing pose (the famous“Statue of Liberty” pose) and not hailing gestures. Our solution, in contrast, analyzes a temporal sequence of images (frames) in order to detect both explicit and implicit hailing scenes. Explicit hailing is decomposed into three visual components, which are the person’s hailing gestures, their relative position to the road, and head orientation. Implicit hailing can be recognized using other contextual information (other than the visual elements). Our solution is also different regarding the perception of the hailing concept.While the work in [15] analyzes car hailing from a computer vision perspective only, our work is inspired by previous studies of taxi hailing as a social interaction between taxi drivers and passengers. This is why we believe that our solution can be easily integrated into a more complete framework of interaction between autonomous taxis and passengers, where hailing is only an aspect of the interaction [14]. Overall, the proposed method yields satisfactory results in relatively realistic settings. However, we noticed that the street-hailing detection fails when the observed scene presents overlapping between vehicles and pedestrians. When this situation occurs, crucial body parts necessary for the accurate detection of hailing gestures are hidden. Therefore, there is still a need for more adjustments of the detection scheme by incorporating more contextual information and by aggregating observations from other monitoring devices, such as other vehicles driving by, on-site CCTV, etc.

## 7. Conclusions

In this paper, we proposed a new method for street-hailing detection based on computer-vision techniques augmented with contextual information. Besides the originality of its deep-learning-based pipeline, our method allows for the detection of both explicit and implicit street-hailing scenes by the integration of visual and contextual information in a logic flow based on a defined scoring scheme. The design of the pipeline was inspired by the feedback collected from 50 experienced taxi drivers that we interviewed in order to understand the intuitive way of identifying prospect street-hailing passengers. We experimentally studied our proposed method using a dataset of urban road video scenes that we collected from a real taxi in the city of Tunis, Tunisia. The study showed that the proposed method achieves satisfactory results with respect to the detection of explicit and implicit street-hailing cases.

Nevertheless, there is still room for future improvement. First, we are currently working on further developing the concept of context. A formal definition of the context is required in order to better model, represent and use contextual information for the detection of both explicit and implicit street-hailing behaviors. Second, the context is also fundamental for the implementation of a more extended street-hailing service stage as a social interaction between Robotaxis and passengers. As we mentioned earlier, hailing recognition is only one part of the hailing interaction, and there is a gap in the state-of-the-art of human–autonomous taxi interaction (HATI) with respect to this aspect. Third, we are collecting more data in order to build a complete dataset dedicated to taxi street-hailing scenes in urban environments that we will use to test and improve the performance of our approach. The dataset will be used to automatically learn the weights of the parameters of the scoring scheme from the real data and shall also exhibit more complex hailing behaviors, as well as monitoring settings, such as different weather, lighting and crowd/traffic conditions.

## Figures and Tables

**Table 1:** Reported performance results of taxi street-hailing detection.

	Accuracy	Precision	Recall	Specificity
Hailing	0.8	0.84	0.84	0.73
Implicit Hailing	N/A	N/A	0.8	N/A
Explicit hailing	N/A	N/A	0.85	N/A

**Table 2:** Visual information score of the explicit hailing scene in Figure 13.

Hailing	Head Direction	Standing Position
50	20	30

**Table 3:** Visual and contextual information scores of the implicit hailing scene in Figure 16.

**Visual** **Information**	**Hailing**	**Head Direction**	**Standing Position**
	10	20	30
**Contextual** **Information**	**Spatiotemporal**	**Weather**	**Event**
	60	30	0

**Table 4:** Visual and contextual information score of the implicit hailing scene in Figure 19.

**Visual** **Information**	**Hailing**	**Head Direction**	**Standing Position**
	0	10	30
**Contextual** **Information**	**Spatiotemporal**	**Meteorological**	**Event**
	30	10	0

## Data Availability

Data used in this work are confidential.
